# Individual Differences in Print Exposure Predict Use of Implicit Causality in Pronoun Comprehension and Referential Prediction

**DOI:** 10.3389/fpsyg.2021.672109

**Published:** 2021-07-26

**Authors:** Elyce Johnson, Jennifer E. Arnold

**Affiliations:** Department of Psychology and Neuroscience, The University of North Carolina at Chapel Hill, Chapel Hill, NC, United States

**Keywords:** pronoun comprehension, implicit causality, individual differences, print exposure, sentence processing

## Abstract

In three experiments, we measured individual patterns of pronoun comprehension (Experiments 1 and 2) and referential prediction (Experiment 3) in implicit causality (IC) contexts and compared these with a measure of participants’ print exposure (Author Recognition Task; ART). Across all three experiments, we found that ART interacted with verb bias, such that participants with higher scores demonstrated a stronger semantic bias, i.e., they tended to select the pronoun or predict the re-mention of the character that was congruent with an implicit cause interpretation. This suggests that print exposure changes the way language is processed at the discourse level, and in particular, that it is related to implicit cause sensitivity.

## Introduction

How does experience with language influence language comprehension? There is extensive evidence that language experience affects lexical and syntactic processing. For example, people tend to produce and understand frequent words and structures more quickly than infrequent words and structures (e.g., Seidenberg, 1985; Trueswell, 1996; Dahan et al., 2001; Wells et al., 2009; Montag and MacDonald, 2015). However, there is more to be known about how language experience affects processing at the discourse level. In the current study, we explore this further by examining how people interpret ambiguous pronouns, and whether their interpretation is influenced by individual differences in how much they are exposed to written language.

Our project focuses on the role of semantic biases in implicit causality (IC) scenarios, namely discourses where causal judgments are important. For example, in *Matt feared Will because he…*, people tend to assume that *he* refers to Will. This interpretation suggests comprehenders are making semantic inferences about who is more likely to be the cause of the fear event. In this example, people tend to expect that the speaker will talk about some action of Will as an explanation of the fearing event, which influences the interpretation of the pronoun *he* (e.g., Kehler and Rohde, 2013). However, this “implicit causality” bias is only one of several constraints known to affect pronoun comprehension. In addition, people tend to interpret the pronoun as co-referential with the grammatical subject (i.e., a subject bias), which here would lead to the assumption that “he” refers to Matt (e.g., Gordon et al., 1993; Brennan, 1995; Nappa and Arnold, 2014). This means that for any given pronoun, comprehenders must weigh different constraints to judge the speaker’s intended meaning.

In this project, we examine whether linguistic experience affects the way comprehenders use implicit causality judgments during pronoun comprehension, and if so, whether it affects early or late pronoun comprehension processes. This question is important because it is well-established that individuals vary in linguistic experience and that experience modulates language processing at multiple levels. However, most of this work addresses language processing at the phonological, lexical, and syntactic levels (e.g., MacDonald, 1993; Saffran et al., 1996; Wells et al., 2009; Farmer et al., 2016). Very little is known about how experience relates to discourse processes like pronoun resolution, and evidence thus far has only demonstrated that print exposure correlates with a syntactically conditioned bias, namely the subject bias (Arnold et al., 2018a, 2019; Langlois and Arnold, 2020). This raises questions about whether language experience can also affect sensitivity to semantic constraints, like implicit causality biases.

In contrast to discourse processing, there is extensive evidence that experience with both written and spoken language affects syntactic processing. There are at least two types of evidence showing this. The first type of evidence is the effect of recent exposure through adaptation and priming. When exposure is manipulated within the context of an experiment, adults can incorporate recent experience with less common structures, exhibiting modified language use according to these patterns. Recent experience facilitates comprehension for less common structures (e.g., Branigan et al., 2005; Wells et al., 2009; Fine and Jaeger, 2013; see also Tooley and Traxler, 2010), and it biases speakers to choose structures that match those they recently heard (e.g., Bock, 1986; Bock and Loebell, 1990; Pickering and Branigan, 1998, 1999; Branigan et al., 2000; Ferreira and Bock, 2006; see also Pickering and Ferreira, 2008). For example, Thothathiri and Snedeker (2008) showed that people tend to interpret temporary ambiguities congruently with syntactic structures they have recently encountered, even for structures that are relatively uncommon in natural language.

A second type of evidence for exposure effects comes from individual difference work. Processing tends to be facilitated for individuals with more language experience or for individuals with exposure to a greater range of words or structures. For example, people with greater exposure to language more easily process and produce low-frequency words and structures than people with less exposure to language (Seidenberg, 1985; Wells et al., 2009; Montag and MacDonald, 2015; James et al., 2018).

Our study instead examines language exposure at the discourse level, focusing on how individuals differ in the mechanisms they use to interpret ambiguous pronouns. Studying individual differences is challenging because experience varies on a number of dimensions. For example, people may differ in either spoken or written language experience, or both. It is difficult to dissociate the effects of the two, which may be correlated. The current study is one of the first to address this question at the discourse level, and because of this, our goal is to focus on a broader question: does at least one type of language experience (here, print exposure) correlate with individual differences in pronoun comprehension?

Our focus is on language comprehension and not reading skills *per se*. We know that reading practice improves reading skills, but reading also provides experience that influences language processes for spoken language use (e.g., Montag et al., 2015; Montag and MacDonald, 2015; Grolig, 2020). Thus, our test of pronoun comprehension is through an auditory language task, which tests the hypothesis that print exposure has effects that extend to spoken language comprehension.

In support of this hypothesis, recent findings suggest that people who read frequently exhibit stronger structural biases in pronoun comprehension than people who do not read as frequently. In Arnold et al. (2018a), print exposure correlated with differences in pronoun comprehension. In this study, a higher score on the Author Recognition Task (ART) was associated with a greater tendency to assign the pronoun to the grammatical subject in a spoken-language comprehension task. In the ART, participants were given a selection of names and asked to identify the real ones, where half of the names were real author names and half were not. Scores on the ART are a gross measure of exposure to literature since a greater recognition of author names is assumed to be correlated with a greater degree of print exposure. This task has been shown to correlate with related measures of reading and reading skills, e.g., verbal comprehension, word identification, word naming, reading speed, and vocabulary (Stanovich and West, 1989; Cunningham and Stanovich, 1991, 1997; Moore and Gordon, 2015).

Arnold et al.’s (2018a) findings suggest that language exposure is instrumental in developing the subject bias for pronoun comprehension. There are several possible explanations for this. One explanation is that reading increases exposure to the most frequent discourse patterns in language. We know that pronouns frequently refer to subjects (Arnold, 1998; Arnold et al., 2018b). People may learn this frequency through exposure, and reading offers one way to increase quantity of exposure. Reading may also provide exposure to a specific type of language that is helpful for learning this bias, namely language that is internally coherent and decontextualized. A second explanation is that reading may provide practice in drawing inferences from the text, which could facilitate a wide range of inferential mechanisms. We discuss these further in the general discussion.

Arnold et al.’s (2018a) results raise a question about how much people can learn from print exposure. We know that the discourse context constrains pronoun comprehension in multiple ways. Does reading facilitate the use of all aspects of the context or just some? Langlois and Arnold (2020) examined this question by asking whether print exposure affects both syntactic and semantic biases in discourses about transfer events. They examined ambiguous pronoun resolution in sentences using transfer verbs (goal/source verbs). For example, participants saw sentences like: *Ana and Liz were playing basketball. Ana threw the ball to Liz, and then she fell down*. Results showed two simultaneous effects – a bias to assign pronouns to the subject character (Ana) as well as an overall goal bias effect, such that people tended to prefer the goal as the referent of the pronoun. However, the goal effect was not related to individual differences, i.e., there was no relationship between ART score and the goal bias. Instead, results showed the same correlation between print exposure and the subject bias as observed by Arnold et al. (2018a). That is, participants with higher ART scores were more likely than those with lower scores to assign the pronoun to the subject, and this trend occurred for both goal-source and source-goal verbs. This seems to suggest that print exposure only affects sensitivity to structural biases. However, this may be due to the relative weakness of the goal bias. Does reading exposure affect the use of stronger semantic biases?

To test this question, here we turn to implicit causality constraints, which are known to strongly influence pronoun comprehension. We test the strength of implicit causality biases by asking people to listen to short passages about an implicit causality scenario, e.g., *Ana and Liz were attending a karaoke party. Ana idolized Liz because she is a great singer*. Our stories use verbs that either put the implicit cause in subject position, as in the previous example (idolize), or in object position (dazzle), e.g., *Ana and Liz were attending a karaoke party. Ana dazzled Liz because she is a great singer*. A question probes their interpretation of the pronoun, e.g., *Who is a great singer?* The rate of selecting the implicit cause character measures the strength of the implicit causality bias for that participant. We ask whether the participant’s print exposure (as measured by the ART task) correlates with the implicit causality bias or whether, instead, it correlates with the rate of selecting the subject character.

If print exposure does correlate with either bias, our next question is why it does so. Several models of pronoun comprehension point to the importance of prediction: as people understand language, they generate probabilistic expectations that a referent will be mentioned. If an ambiguous pronoun is encountered, there is a bias toward the expected referent (Arnold, 1998, 2001; Kehler and Rohde, 2013; see also Hartshorne et al., 2015 for a similar idea). These expectations are guided by coherence relations. Coherence relations describe a relationship between two clauses and can influence referential expectations (Kehler et al., 2008; Kehler and Rohde, 2013). While the connector word is not necessary to form a coherence relation, the choice of a connector word (e.g., *and then*, *so*, and *because*) greatly influences the perception of the coherence relation. For example, the expectation for a causal continuation is strengthened when *because* links the first and second clause. For that reason, many implicit causality studies (as well as the current study) make this relation explicit by using the word *because*, which signals an explanation relationship and prompts an expectation to continue speaking about the cause (Ehrlich, 1980; Au, 1986; Stevenson et al., 1994; Kehler and Rohde, 2013). This expectation is integrated with later bottom-up processes driven by the pronoun and subsequent material, which ensure that the pronoun gender and number match the referent and that the post-pronominal material is consistent with the assumed referent (Brown and Fish, 1983; Au, 1986; Gordon et al., 1993; Brennan, 1995; LaFrance et al., 1997; Corrigan, 2001, 2002, 2003).

A related issue emerges in a debate about the time course of implicit causality effects on pronoun comprehension. The expectation-driven (or focusing) account asserts that verb bias has early effects, such that before any disambiguating information is reached, the implicit cause becomes more accessible and is considered a likely candidate for re-mention (McDonald and MacWhinney, 1995; Long and De Ley, 2000; Koornneef and Van Berkum, 2006; Pyykkönen and Järvikivi, 2010; Cozijn et al., 2011; Järvikivi et al., 2017). By contrast, the integration account posits that the implicit cause effect does not occur until later when disambiguating information is integrated (Garnham et al., 1996; Stewart et al., 2000).

While the focus of this debate is the timing of processing, it may be related to the information available at different points in a discourse. Consider a sentence like *Ana and Liz were attending a karaoke party. Ana idolized Liz because she is a great singer*. Readers are likely to assume that Liz is the referent of “she,” potentially for two reasons. Liz is the implicit cause of the idolizing event, which may lead readers to anticipate future mention of Liz as soon as they encounter the verb *idolize*, and this prediction would be supported by the word *because*. We consider these “early” sources of information. In addition, the second clause provides a plausible explanation for the idolizing event, namely that someone is a great singer. Given that the possession of skills is frequently seen as an explanation for being idolized and not a reason to idolize someone else, this leads to the inference that *she* probably refers to Liz. We consider these inferences to be “late” sources of information.

Our question here is whether print exposure correlates with early expectation-driven processes or only later inference-driven processes. We test this in two ways. First, we use two different tasks for testing print exposure. In the first task (Experiment 1), participants hear short stories where the subordinate clause ends with the novel word “dax,” for example, *Ana and Liz were attending a karaoke party. Ana idolized Liz because she is a dax* (task-based on Hartshorne and Snedeker, 2013). They are then asked, *Who is a dax?* and in this example, have a choice between Ana or Liz. In the second task (Experiment 2), participants hear short stories like *Ana and Liz were attending a karaoke party. Ana idolized Liz because she is a great singer*. In this task, they are asked *Who is a great singer?* Our task only collects offline judgments (i.e., after comprehension has finished), which means we cannot pinpoint when the judgments are being made. Nevertheless, by using a novel word (dax), we limit the possible constraints from post-pronominal material. This means we can assume that responses are based mostly on early information, i.e., that which occurs prior to the pronoun. Critically, since neither the pronoun nor the novel word carries any disambiguating information, an implicit cause interpretation would have to be made just on the information available from the verb and the clausal connector, i.e., *because*.

Second, in Experiment 3, we test whether print exposure correlates with predictions about who will be mentioned next in the absence of a pronoun. We provide participants only with the initial fragment, e.g., *Ana and Liz were attending a karaoke party. Ana dazzled Liz because …*, and ask them to judge which character is most likely to be mentioned next. If the next-mention judgments follow the same pattern as the pronoun comprehension judgments, it will suggest that the effects stem from information available during early processing prior to the pronoun. Supplementary Material for all experiments are available at http://arnoldlab.web.unc.edu/publications/supporting-materials/johnson-arnold/ and data are available at https://osf.io/fuzp2/.

## Experiments 1 and 2

### Methods

#### Participants

Both Experiments 1 and 2 were administered *via* Amazon Mechanical Turk. All participants were native English speakers, at least 18 years of age. All participants were paid for participation. Our target sample was 60 participants in each experiment, following the sample size used in other studies that tested the effect of print exposure (e.g., Arnold et al., 2018a). Participants were excluded if more than 1/3 of their responses on the ART were incorrect because it signals that the participant was ignoring the instructions to not guess. Second, participants were excluded if they answered fewer than 75% of the filler questions correctly because this signals that they were not consistently paying attention. These measures are especially important given the use of online participant recruitment to restrict our sample to participants who were paying attention. We replaced the excluded participants to meet our target sample size of 60 in each experiment.

In Experiment 1, 21 were replaced (16 for guessing on ART, three for low filler accuracy, and two for both). In Experiment 2, 29 were replaced (seven for guessing on ART, seven for low filler accuracy, and 15 for both). Our final sample for analysis was 60 participants in each experiment.

#### Procedure

On Amazon Mechanical Turk, participants were directed to a link for the Qualtrics survey. At the beginning of the survey, participants were instructed to read and indicate that they agreed to the consent form. They were also informed that there would be several check questions in the survey and that if they had too many incorrect responses, they would be dismissed from the survey without pay. Participants then completed the demographic questions, the main task, and then the ART in that order. At the end of the experiment, they received an end-of-survey message thanking them for their time and a randomly generated number to record on the Amazon Mechanical Turk site for payment.

#### Materials and Measures

Both experiments were designed in Qualtrics. In the main task, participants heard an audio recording of the story and saw pictures of the mentioned characters. After listening to the audio clip, they answered two multiple choice comprehension questions about the story’s content. Following the main task, they completed the ART.

The main task consisted of two practice items and 12 target items. Experiment 1 also had eight filler items, while Experiment 2 had 12 fillers. Sample target stimuli are shown in Table 1. For both experiments, stimuli included a context sentence that introduced two same-gender characters in a conjoined subject NP, followed by a target sentence that used a verb with a strong implicit causality (IC) bias, where the two characters fell in subject and object positions, e.g., *Ana and Liz were attending a karaoke party. Ana dazzled Liz because she …*. All target items used same-gendered characters, making the pronoun ambiguous. The two experiments were almost identical, but Experiment 1 used the novel word *dax* in the final clause of all sentences (…*because she is a dax*), while Experiment 2 used a real ending (e.g., …*because she is a great singer*). Thus, in Experiment 1, the final clause was semantically ambiguous with respect to the pronoun identity, whereas in Experiment 2, the final clause was semantically more consistent with the implicit cause interpretation of the pronoun. Stories were followed by two questions; the critical question probed the interpretation of the pronoun (*Who is a dax?* or *Who is a great singer?*).

**Table 1:** One target item appears across two lists, differentiated by verb type.

Experiment	Condition	Context sentence	Target sentence	Critical Question
1	Object-biased	Ana and Liz were attending a karaoke party	*Ana idolized Liz because she is a dax.*	Who is a dax? (Ana/Liz)
1	Subject-biased	Ana and Liz were attending a karaoke party	*Ana dazzled Liz because she is a dax.*	Who is a dax? (Ana/Liz)
2	Object-biased	Ana and Liz were attending a karaoke party	*Ana idolized Liz because she is a great singer.*	Who is a great singer? (Ana/Liz)
2	Subject-biased	Ana and Liz were attending a karaoke party	*Ana dazzled Liz because she is a great singer.*	Who is a great singer? (Ana/Liz)

The critical items followed the same manipulations and experiment design for the two experiments. The 12 target items appeared in two versions: one in which the second sentence contained a subject-biased IC verb, and one in which the second sentence contained an object-biased IC verb. Thus, for the 12 target stories, we used a total of 24 verbs, 12 subject-biased and 12 object-biased. The two versions of the target items were the same in that they shared the same context sentence. Verb type was manipulated within-subject such that each participant heard six object-biased items and six subject-biased items. Thus, two lists were created with one version of an item per list (Table 1). As a control variable, there were two versions of each list in forward and backward order, for a total of four lists, for all items see Appendix A in Supplementary Material.

Lists were matched for average verb bias, verb frequency, and verb valence rating. Verbs were selected from experiment 2 of Hartshorne and Snedeker (2013), where verb bias was recorded as the percentage of participants who selected the object as the referent of the pronoun. The verbs selected for this study had either an object bias of greater than 70% (object-biased condition) or an object bias of less than 30% (subject-biased condition). Verbs were also classified according to their placement in the verb classification of Levin (1993) in which all verbs are either of the class 31.1 (amuse verbs), which tend to be subject-biased, or of the class 31.2 (admire verbs), which tend to be object-biased. Measures of frequency for the verbs were collected *via* the SUBTLEX-US word frequency database (Brysbaert and New, 2009), and valence ratings came from Warriner et al. (2013). See Appendix A in Supplementary Material for biases, ratings, and frequencies.

Filler items were similar to the target items in that they consisted of two sentences, a context sentence and a two-clause sentence, but unlike the targets, they were disambiguated by different gender characters, and they were not implicit cause sentences. In Experiment 1, the eight fillers took the form *X is doing something with Y*, which we term the “joint action” structure, e.g., *Liz and Will were on vacation*. *Liz watched TV with Will because she is a dax*. In Experiment 2, where the final clause was consistent with a single interpretation of the pronoun, we sought to increase variation across responses so that participants would not fall into a pattern of responding. We, therefore, used six joint action fillers, plus another six fillers with transfer verbs, e.g., *Will and Ana were at McDonald’s*. *Will took the fries from Ana because she was full*.

There were two types of questions for both target and filler items. Critical questions always asked *who is a dax?* (Experiment 1) or, e.g., *who is a great singer?* (Experiment 2). These questions tested how the participant interpreted the pronoun. Critical question answer choices were always the two characters in the sentence, and content question answer choices were always yes/no. The other question was a content question that also functioned as an attention check question for filler items. These questions asked about information in the second clause of the sentence. For example, in the sentences, *Liz and Matt were studying for an exam. Liz went to the library with Matt because he is a dax*, the content question asked *Did Liz go to the library with Matt?* The answer choices were either *yes* or *no*. Both the critical question and the content question were formatted similarly in that they were multiple choice questions with two answer options. The content question responses for eight of the filler items were used to make sure that participants were reading the sentences; if participants missed more than 25% (i.e., three items), they were automatically dismissed from the survey and not paid. In addition, we checked all filler content question responses and replaced any participants who missed more than 25% of the total. For all questions, the order in which answers appeared in the multiple-choice selection (top/bottom) was counterbalanced for all questions so that yes/no answer options appeared equally in each position and character name options (e.g., Liz/Ana) appeared equally in each position. Whether they received a critical question or a content question first or second was also counterbalanced.

**Figure 1:**
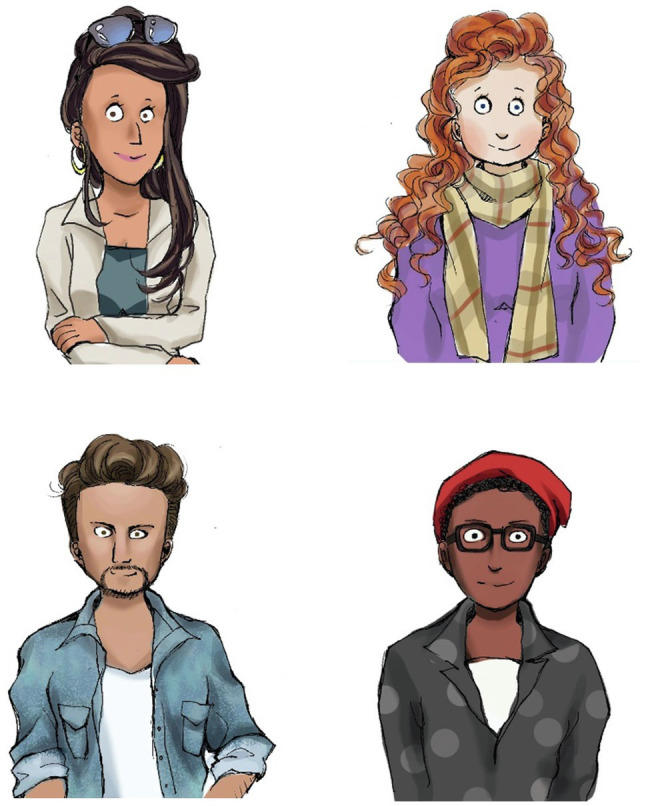
There are four characters used in this experiment, these are the images that correspond to each character. Top left – Ana, Top right – Liz, Bottom left – Matt, and Bottom right – Will. These pictures are copyrighted to Chan et al. (2018), and used by permission.

For all items (target and filler), there were four characters: Ana, Liz, Will, and Matt (Figure 1). For the images that accompanied the audio, the first-mentioned character always appeared on the left side, and the second-mentioned character always appeared on the right side. Across all stimuli, each of the four characters occurred equally in subject and object position. See Appendix A in Supplementary Material for a transcription of the stimuli. Audio and images were presented on the same page, followed by two comprehension questions on the next page with two forced-choice answer choices per question. Audio recordings auto-played and the button to advance to the next page did not appear until 5 s after the duration of the audio to ensure that participants listened. Each question appeared on a separate page.

Prior to the main task, participants answered background questions about themselves, their language experience, and socioeconomic status (SES). Following the main task, participants completed the ART. We used a modified version of this task developed by Peter Gordon, which was based on previous versions of the task (Stanovich and West, 1989; Acheson et al., 2008; Moore and Gordon, 2015; See Appendix B in Supplementary Material). Participants saw an array of 126 names, 63 author names, and 63 foils. Participants were asked to select only the names that are author names, and they were instructed not to guess. Their ART scores were calculated based on the names they selected. They received one point for each correct name (+1) and were penalized for selecting an incorrect name (−1). The number of incorrect name selections was used as a metric to identify participants who guessed or selected names without reading; participants whose responses included more than 1/3 incorrect names were not included in the analysis.

### Analytical Approach

We analyzed the data with a mixed-effects logistic regression using SAS proc. glimmix with a binary distribution and a logit link. The dependent measure was whether the participant selected the grammatical subject as the referent of the pronoun or not. It was coded as a binary measure with 1 for a grammatical subject selection and 0 for no grammatical subject selection. Predictors included verb bias, which was effect coded (0.5 = subject-biased; −0.5 = object-biased), and ART scores, which were grand-mean centered. All models used random intercepts for both participant and item and maximal random slopes except where noted.

For each experiment, we first performed a baseline model that included only the manipulated verb bias to assess its effect. Then for our primary analysis, we added print exposure scores and their interaction with verb bias.

### Results

#### Experiment 1 Results

Participants were more likely to identify the subject as the referent of the pronoun in the subject-biased condition (64.4%, *SE* = 0.042) than in the object-biased condition (11.4%, *SE* = 0.022). The baseline model confirmed that this effect of verb bias was significant (*β* = 2.987, *SE* = 0.350, *t* = 8.53, *p* < 0.001).

**Figure 2:**
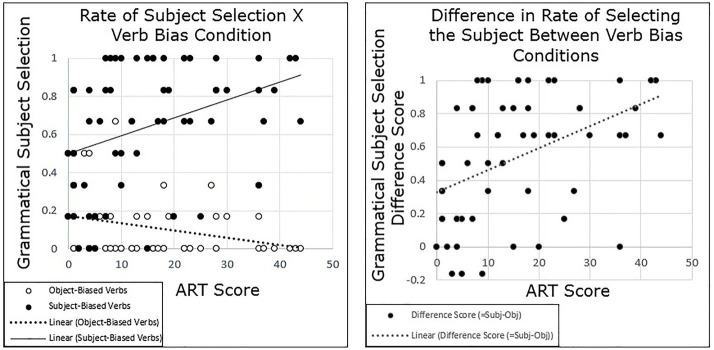
Experiment 1 results: participants with higher Author Recognition Task (ART) scores show a stronger semantic bias than those with lower ART scores. **(Left panel)** Each dot represents the participant’s average rate of selecting the subject in each condition, such that there are two dots shown per participant. **(Right panel)** This graph depicts differences scores, such that each participant’s average rate of subject selection in the object-biased verb condition was subtracted from their average rate of subject selection in the subject-biased verb condition. This demonstrates the variability of the semantic bias for each participant.

Our primary question of interest was whether individuals’ pronoun interpretation would vary by their performance on the ART and whether this variation would be correlated with variation in an implicit cause bias, a subject bias, or both. Participants who scored higher on the ART were more likely to select the subject for the subject-biased verbs, and more likely to select the object for the object-biased verbs, resulting in a greater effect of verb bias than for participants who scored lower on the ART (Figure 2). In short, participants with greater print exposure showed a stronger implicit causality bias than participants with lower print exposure.

To test the significance of this pattern, grand-mean centered ART scores were added to the model, as well as the interaction between ART and verb bias. Results again showed a main effect of Condition (*β* = 2.903, *SE* = 0.333, *t* = 8.71, *p* < 0.001). There was no main effect of ART (*p* = 0.702), but there was a significant interaction between ART and verb bias (*β* = 0.091, *SE* = 0.0261, *t* = 3.49, *p* < 0.001), such that as ART score increased, participants were more likely to follow a semantic bias.

#### Experiment 2 Results

Again, participants were more likely to identify the subject as the referent of the pronoun in the subject-biased condition (78.3%, *SE* = 0.032) than in the object-biased condition (8.3%, *SE* = 0.018). The baseline model again revealed a main effect of verb bias (*β* = 4.074, *SE* = 0.397, *t* = 10.26, *p* < 0.001).

**Figure 3:**
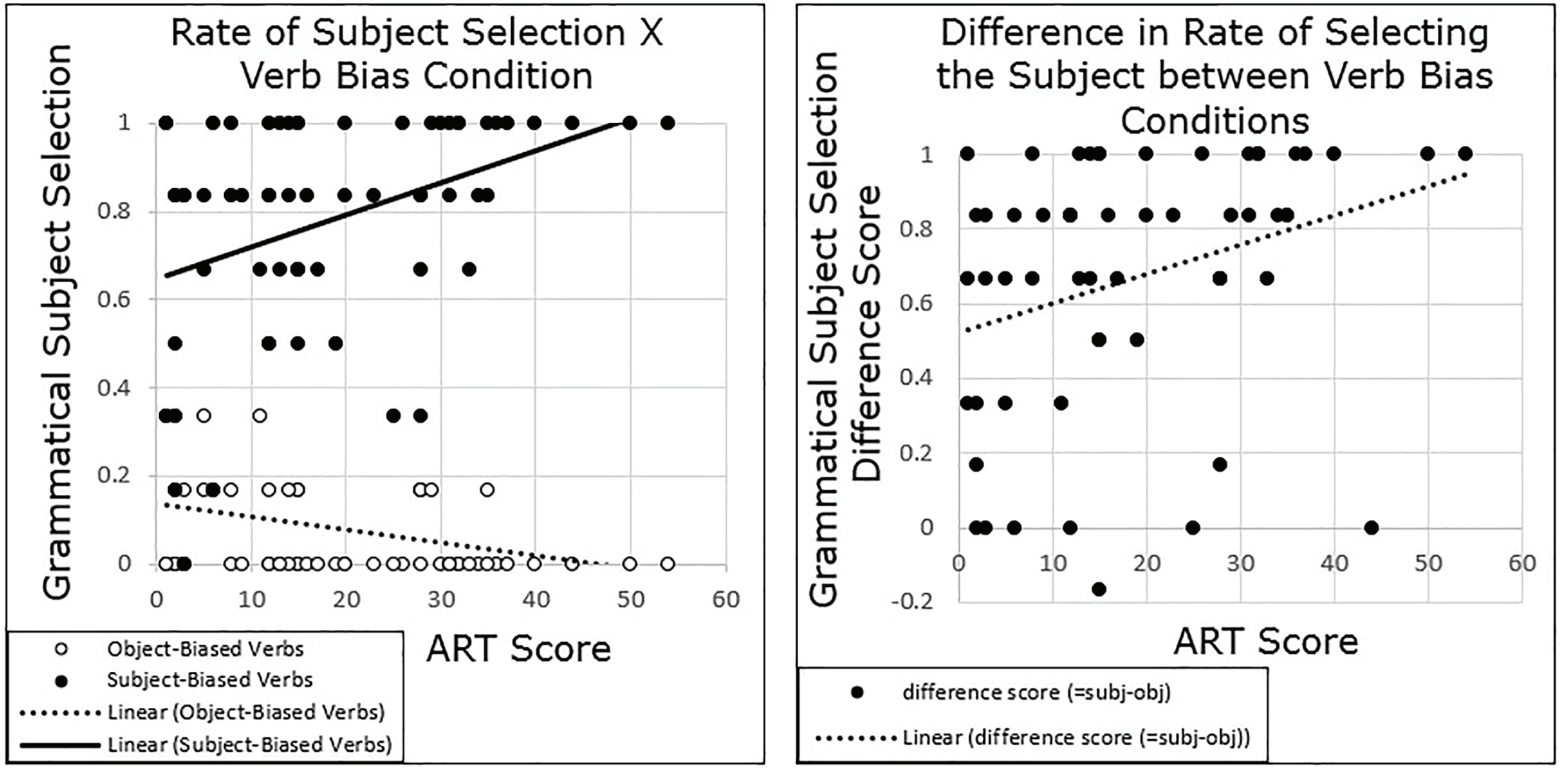
Experiment 2 results: participants with higher ART scores show a stronger semantic bias than those with lower ART scores.

As in Experiment 1, we also found that participants with higher ART scores exhibited a stronger implicit causality bias for pronoun comprehension compared with participants with lower ART scores. Grand-mean centered ART scores were added to the model, as well as the interaction between ART and verb bias. Results again revealed a main effect of Condition (*β* = 4.113, *SE* = 0.388, *t* = 10.6, *p* < 0.001). There was no main effect of ART (*p* = 0.823), but there was a significant interaction between ART and verb bias (*β* = 0.105, *SE* = 0.029, *t* = 3.59, *p* < 0.001),^1^ such that as ART score increased, participants were more likely to follow a semantic bias (Figure 3).

Our analyses were aimed at assessing individual differences in the implicit causality bias. Nevertheless, we note that in both experiments, participants did not show a subject bias, as some other studies have reported (e.g., Stevenson et al., 1994; Kehler et al., 2008). Instead, they showed a general object bias. Across all items, the average subject response was 37.9% for Experiment 1 and 43% for Experiment 2.

### Experiment 1 and 2 Discussion

Experiments 1 and 2 tested individual differences in the interpretation of pronouns, examining causal contexts where the verb bias is expected to guide interpretations. We found that individual performance on a print exposure task was correlated with individual differences in the pronoun task: participants with higher ART scores were more sensitive to the verb bias than participants with lower ART scores.

Notably, the interaction between ART and verb bias was consistent across Experiments 1 and 2, even though the pronoun-clause was ambiguous in Experiment 1, but not in Experiment 2. In Experiment 1, the nonword “dax” provided no information about the pronoun referent, requiring participants to rely only on the pre-pronoun context (verb bias and “because” connector) for interpretation. In Experiment 2, the post-pronoun context provided a continuation that was semantically consistent with the verb bias, so in theory, participants could use either the pre-pronoun context or the post-pronoun context or both. Our findings suggest that print exposure is correlated with participants’ sensitivity to the pre-pronoun context, which was available in both experiments. The fact that we see similar implicit cause effects occur in each experiment suggests that there is something over and above the information in the last clause of the implicit cause sentences that leads to an implicit clause bias. However, we cannot disentangle the importance of verb bias from coherence relation. Our stimuli used the word “because,” which prompts an expectation of information about implicit cause (Au, 1986; Stevenson et al., 1994; Kehler and Rohde, 2013; Guan and Arnold, 2021). Thus, the observed individual differences may relate to the use of the verb and/or the use of the coherence relation as signaled by the word “because.”

We found no subject bias, that is, no general preference to select the subject character as the referent of the pronoun. The lack of a general subject bias contrasts with other studies with similar stimuli (e.g., Stevenson et al., 1994; Kehler et al., 2008). Instead, we found an object bias, similar to Hartshorne and Snedeker (2013). This raises an interesting question as to why the historically found subject bias is not present in this study. In any case, in principle, we could have still seen individual variation in the degree of relative subject bias, yet we did not. Critically, ART scores did not correlate with the subject bias overall, which contrasts with previous findings (Arnold et al., 2018a; Langlois and Arnold, 2020). We will take this up in the general discussion.

Together, the results of Experiments 1 and 2 showed that people differ from each other in how strongly they follow the implicit causality bias. Similar results across experiments suggest that individual differences stem from information up until the pronoun, but not including the post-pronominal information. This suggests that individuals may differ in the degree to which they use the context to make predictions about the upcoming discourse. Participants who read frequently may be better equipped to use the semantic context to generate predictions about likely upcoming mentions. If frequent readers are more consistent in their expectations that the implicit cause will be mentioned, they would also be likely to transfer that prediction onto the interpretation of the pronoun.

We test this idea explicitly in Experiment 3 with the use of a metalinguistic prediction task. We know that verb bias affects predictions: when people read a fragment like *Ana admired Liz*, they judge that *Liz* should be more likely to be mentioned than *Ana* (Guan and Arnold, 2021; Weatherford and Arnold, 2021). Our question here is whether this bias varies across individuals. Do frequent readers also show a stronger sensitivity to implicit causality when making predictions about who will be mentioned next?

## Experiment 3

### Methods

#### Participants

Seventy-nine native speakers of English participated through Amazon Mechanical Turk. A total of 19 participants were excluded (two for incorrect ART, 14 for incorrect fillers, and three for both incorrect ART and fillers). We replaced the excluded participants to meet our target sample size of 60 participants. Exclusion criteria were the same as in Experiments 1 and 2.

#### Procedure

The procedure for Experiment 3 was the same as for the first two except that the ART came before the main task instead of after. We shifted the position because we thought this might reduce the number of ART exclusions, which it did, compared to Experiments 1 and 2. Our reasoning was that we thought participants were either weary or rushing to finish the ART at the end, especially given that performance on the ART would not cause them to be kicked out of the survey or affect them receiving payment.

#### Materials and Measures

The third experiment used the same critical stimuli as for the first two experiments, but the audio was cropped in Praat (Boersma, 2001) prior to the pronoun. This allowed us to assess how participants were making predictions given the implicit cause verb and the word *because*. Our key question was, “Who is likely to be mentioned next?” Participants chose between the names of the two characters.

We used the audio from Experiment 1 for the stimuli, although the pre-pronoun texts were identical across Experiments 1 and 2, so this choice was not critical. All target items were cropped right after *because*. For example, participants might hear *Ana and Liz were attending a karaoke party. Ana idolized Liz because—*. We only used four of the original filler items from Experiment 1 in order to shorten the experiment. Predictions are less constrained than meaning-based interpretations, and we expected that participants would be likely to fatigue if given a longer experiment.

The main task consisted of two practice items, 12 target items, and four filler items. The filler items were the same as in Experiment 1, but we included part of a person’s name before the truncation point, e.g., *Will and Ana were at the gym. Will ran a mile with A—*. Thus, participants heard part of the next mentioned character, but not the full name. Participants were instructed to choose the person who was partially mentioned as the person to be mentioned next. Just as in the first two experiments, the 12 target items appeared in two versions: one in which the second sentence contained a subject-biased verb and one in which the second sentence contained an object-biased verb, forming two lists, see Table 2. A total of 24 verbs were used in this study, 12 per list (six subject-biased and six object-biased).

**Table 2:** One target item appears across two lists, differentiated by verb type.

List	Condition	Context sentence	Target sentence
1	Object-biased	Ana and Liz were attending a karaoke party.	Ana idolized Liz because –
2	Subject-biased	Ana and Liz were attending a karaoke party.	Ana dazzled Liz because –

Following the audio clip, participants answered two questions. Critical questions always asked *who is likely to be mentioned next?* The other question was a content question that also functioned as an attention check question for both target and filler items. There were two types of content questions: in the target items, the content question only asked about one character e.g., in the sentences, *Liz and Ana were working out at the gym*. *Liz loathed Ana because—* participants saw a content question like, *What did Liz do?* In this example, the answer would be *loathed Ana*. In filler items, the content question always asked about both characters, e.g., in, *Will and Ana were at the gym. Will ran a mile with A—*, the content question would ask *What did Will and Ana do?* and the answer would be *ran a mile*.

The demographic questions and ART were identical to Experiments 1 and 2.

### Analytical Approach

Data analysis was the same as for Experiments 1 and 2, except that the dependent measure was whether the participant selected the grammatical subject as the character likely to be mentioned next. It was coded as a binary measure with 1 for a grammatical subject selection and 0 for no grammatical subject selection.

### Results

Participants were more likely to identify the subject as likely to be mentioned next in the subject-biased condition (67.5%, *SE* = 0.042) than in the object-biased condition (17.78%, *SE* = 0.034). A baseline statistical model, testing only the predictor for verb bias, confirmed that there was a significant main effect of verb bias (*β* = 2.79, *SE* = 0.317, *t* = 8.80, *p* < 0.001).

**Figure 4:**
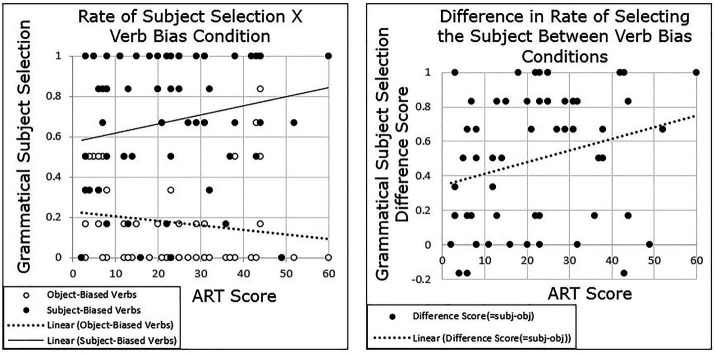
Experiment 3 results: participants with higher ART scores show a stronger semantic bias than those with lower ART scores.

The primary question of this experiment was whether individuals’ prediction of next mention would vary by their performance on the ART and whether this variation would be correlated with variation in an implicit cause bias, a subject bias, or both. Grand-mean centered ART scores were added to the model, as well as the interaction between ART and verb bias.^2^ Results revealed a main effect of Condition (*β* = 2.844, *SE* = 0.314, *t* = 9.05, *p* < 0.001) and showed no main effect of ART (*p* = 0.857), but there was a significant interaction between ART and verb bias (*β* = 0.047, *SE* = 0.022, *t* = 2.14, *p* = 0.036), such that as ART score increased, participants were more likely to follow a semantic bias. Participants who scored higher on the ART showed a greater disparity in grammatical subject prediction than did those who scored lower on the ART. Since grammatical subject response was the dependent variable, this is represented by an increase in the selection of the grammatical subject as ART score increased in the subject-biased condition, and a decrease in the selection of the grammatical subject as ART score increased in the object-biased condition (Figure 4).

Again, participants displayed an overall object bias. When the verb was object-biased, participants predicted the character in the object-position (82.2%) would be mentioned next more often than they predicted the character in the subject-position (67.5%) would be mentioned next when the verb was subject-biased.

### Experiment 3 Discussion

Results from Experiment 3 paralleled findings from Experiments 1 and 2: people followed an implicit causality bias when asked to make judgments about who is likely to be mentioned next. Moreover, print exposure influenced the consistency of referential predictions across individuals. People who read more were more likely to predict an implicit cause in a task that did not involve pronoun interpretation.

These results support the conclusion that the results in Experiments 1 and 2 were driven by the information available up until and including the word *because*, and not inferences from the information after the pronoun. Here, we saw that predictions before the pronoun were influenced by both verb bias and print exposure. Likewise, in Experiment 1, we saw that pronoun interpretation was influenced by verb bias and print exposure, even though the post-pronominal information was not informative. Together, they suggest that people form referential predictions by the time they finish reading *because* and that these predictions guide pronoun comprehension.

## General Discussion

The current study explored whether there were individual differences in ambiguous pronoun resolution in the context of implicit causality verbs, as well as in predictions of likelihood of mention in the same linguistic context. The results of both Experiment 1 and 2 showed that participants’ pronoun resolution varied by their scores on the ART, a proxy for print exposure (see Figures 2, 3). Participants with higher ART scores tended to show a stronger semantic bias in both experiments, measured as a larger difference between the subject-biased verbs and object-biased verbs, compared with participants with lower ART scores. Similarly, the results of Experiment 3 showed that participants’ prediction of who is likely to be mentioned next varied by scores on the ART, and it did so in the same pattern as for Experiments 1 and 2 (see Figure 4). Participants with higher ART scores demonstrated a stronger semantic bias, and those with lower scores demonstrated a weaker semantic bias.

These findings are important for several reasons. First, they add to the body of research that shows that there are individual differences in pronoun comprehension (Daneman and Carpenter, 1980; Francey and Cain, 2015; Arnold et al., 2018a; Langlois and Arnold, 2020), and they contribute to the growing body of literature showing individual differences in pronoun resolution as a function of print exposure (Arnold et al., 2018a, 2019; Langlois and Arnold, 2020). Critically, our study extends these findings by showing that individual differences in print exposure also influence pronoun resolution in implicit causality contexts.

Results from Experiment 3 also inform the body of literature that explore the time course of referential processing (e.g., McKoon et al., 1993; Greene and McKoon, 1995; Long and De Ley, 2000; Koornneef and Van Berkum, 2006; Järvikivi et al., 2017). Although we did not test time course of processing *per se*, we showed that predictions congruent with a semantic bias can be made prior to any reference in the form of a pronoun or otherwise. In addition, those predictions varied in line with individual performance on the ART. This raises questions about whether print exposure may also relate to differences in the time course of processing.

Previous work has demonstrated that, on average, people tend to expect that the implicit cause will be mentioned. In a sentence fragment like *John admired Bill because he…* people are likely to complete this sentence in such a way that the pronoun refers to Bill, the implicit cause (Garvey and Caramazza, 1974; Garvey et al., 1976; Brown and Fish, 1983; Stevenson et al., 1994; Kehler et al., 2008), and in a complete sentence like *John admired Bill because he is great father*, participants consistently show an expectation for the implicit cause, whether they are probed at different points within the sentence or timed in a self-paced reading task (McDonald and MacWhinney, 1995; Garnham et al., 1996; Long and De Ley, 2000; Koornneef and Van Berkum, 2006). So, the question then is: what is it about print exposure that contributes to the variation we see in how people apply implicit cause cues to predictions and interpretations in pronoun resolution?

We consider two accounts for how print exposure might contribute to pronoun and prediction judgments in implicit causality sentences. The Referential Frequency interpretation is that perhaps adults who have had more print exposure than other adults have encountered more instances of implicit causality scenarios, so they have a more robust dataset from which to learn about the frequency of re-mentioning the implicit cause. Evidence from a text analysis shows that in sentences like the ones in our experiment, speakers tend to re-mention the implicit cause more than the other character (Guan and Arnold, 2021). That is, the relative frequency of implicit cause re-mention is high in these types of sentences.^3^ Through exposure to this pattern, language users may learn that that the implicit cause is a more likely continuation and learn to focus attention on it before any anaphoric reference is mentioned. This interpretation is consistent with other proposals that as we are exposed to language over a lifetime, we become predisposed to common patterns of reference that in turn influence our expectations (Arnold, 1998, 2001, 2010). These patterns could be learned through either spoken or written language. On this view, print exposure matters because it is one type of language experience, and it adds to one’s overall lifetime experience with language. People who read more may receive a higher quantity of input than people who read less, thus increasing their exposure to the typical patterns of reference in language. It may additionally be the case that reading can provide a particularly useful type of language input that is helpful for learning about what patterns of reference are more frequent (for further discussion of this point, see Arnold et al., 2018a). On the other hand, individual differences in print exposure may also be correlated with individual differences in other language domains, so we cannot conclude that written language is the only source of individual variation. Nevertheless, the robust effect of print exposure is consistent with the idea that language exposure may help people learn discourse statistics.

Alternatively, the Semantic Inference account might explain our finding by building on the idea that semantic constraints matter for predicting the re-mention of a particular referent. For example, Kehler et al.’s (2008) model (see also Kehler and Rohde, 2013; Rohde and Kehler, 2014) suggests that people keep track of the likelihood that each referent will be mentioned and that they do so on the basis of semantic representations. In their model, the semantic constraints of implicit causality sentences increase the probability that the implicit cause will be re-mentioned. For example, if you hear *Ana admired Liz because…* as the listener you are likely to expect the speaker to mention the person who is most likely the cause of the admiration. An open question is whether these semantic biases are generated each time people read a sentence or whether they are learned and stored in memory. While the Kehler/Rohde model does not make this explicit, its reliance on the semantic representation is consistent with the idea that listeners make semantic inferences anew for each situation (see also Hartshorne et al., 2015). Conceivably, print exposure could matter because it is related to one’s ability to make semantic inferences on a case-by-case basis. Perhaps high print exposure people are better able to use the information at hand to make inferences about likelihood of mention because they have more opportunities to practice doing this. This is in line with findings showing that language skills are stronger for individuals with greater language exposure (Stanovich and West, 1989; Cunningham and Stanovich, 1991; Wells et al., 2009; Mani and Huettig, 2014; Montag and MacDonald, 2015). Language comprehension involves the ability to make causal judgments and inferences that facilitate local and global coherence of discourse ideas (Graesser et al., 1997). Research shows that the ability to preserve the links between the influx of new information and relevant older information requires higher-order cognitive processes (Graesser et al., 1997) and that there are individual differences in both print exposure and language ability that predict one’s ability to do so (Osana et al., 2007; Dai et al., 2019).

While here we focused on print exposure, a related question is whether individual differences in the use of implicit causality inferences relate to individual variation in reading skill. Given that the ART is correlated with measures of reading skill (Moore and Gordon, 2015), we might expect that reading skill also correlates with pronoun comprehension. Indeed, research suggests that it does. For example, Francey and Cain (2015) showed that children with poor reading comprehension skills were less skilled at resolving pronouns in gender-ambiguous cases than children with better reading comprehension skills. Their stimuli required implicit cause inferences by using *because* as the clause connector, e.g., *Michael handed a thank you note to Adrian, after the party,* and *because he was polite*. Each story was followed by a pronoun comprehension question such as *Who was polite?* They found no effects of skill when the gender cue was unambiguous (i.e., two different-gendered characters), but when the pronoun was ambiguous by gender, performance suffered for children with lesser reading skills. A similar conclusion comes from Long and De Ley (2000). They used a probe task to assess processing of implicit causality verbs in two-clause sentences containing a congruent explanation in the subordinate clause. They found that skilled readers were faster at identifying names congruent with the bias than less skilled readers and were significantly more accurate in their responses to the probes. Results also showed that less skilled readers only showed an effect of implicit causality on pronoun resolution after they had integrated information from both clauses.

Thus, previous studies show that using implicit causality information also correlates with reading skills (see also Daneman and Carpenter, 1980). But critically, these studies tested comprehension in a reading task. It is not surprising that reading skill leads to better performance on a reading task. Thus, previous findings could be interpreted as evidence that better readers are better able to extract information from the written page. The current results provide a critical extension to previous work by demonstrating that print exposure scores correlate with performance on a spoken language comprehension task. This demonstrates that print exposure affects interpretation of pronouns in a way that is not modality specific. Our work also demonstrates that individual differences in pronoun comprehension are specifically linked to differences in prediction inferences.

There are still several unanswered questions about how print exposure relates to pronoun comprehension and prediction. We have suggested two possible explanations for why individuals differ in their usage of implicit causality for pronoun comprehension, the Frequency account, and the Semantic Inference account. Further work is needed to test these accounts. A potential concern for the Semantic Inference account is that it does not offer an obvious explanation for why people with high print exposure tend to follow the subject bias more consistently in other experiments. Arnold et al. (2018a, 2019) found this pattern in structures like *Ana was cleaning up with Liz. She…*, and Langlois and Arnold (2020) found this pattern in sentences using transfer verbs (goal/source verbs). Given that reference to the prior subject is a frequent occurrence for both verb types (Arnold, 1998, 2001; Arnold et al., 2018b), the Frequency account can explain this by suggesting that print exposure strengthens representations about which referential patterns are most likely.

On the other hand, a potential concern for the Frequency account is that print exposure has different effects for different semantic biases. In the three experiments reported here, print exposure increased reliance on a semantic bias, implicit causality. But for transfer verbs, Langlois and Arnold (2020) found that print exposure was unrelated to a different semantic bias, the goal bias. Given that goals do tend to be mentioned more often than sources (Arnold, 2001), one would expect print exposure to correlate with this bias. On the other hand, the goal bias is weak. Perhaps stronger semantic biases are more likely to be learned from observing frequencies in the input, or perhaps individual differences are easier to detect for stronger biases. Further work is needed to understand whether print exposure correlates only with some types of semantic biases and how these biases are learned.

While the precise mechanism behind individual differences needs further exploration, the current study makes a valuable contribution to the field by demonstrating that print exposure correlates with the use of implicit causality for both pronoun interpretation and referential prediction judgments. We have shown that this effect is not limited to reading but also affects spoken language comprehension. Our findings build on the results of previous studies that showed that implicit causality makes one referent more predictable (Kehler et al., 2008; Fukumura and van Gompel, 2010; Kehler and Rohde, 2013; Guan and Arnold, 2021), and demonstrate that these judgments themselves are modulated by print exposure. Both our prediction findings (Experiment 3) and our findings from the ambiguous-word experiments (Experiment 1) reveal that these biases emerge from information in the first sentence.

We also looked at whether individuals’ pronoun resolution would vary with respect to their SES. We wanted to determine whether the observed ART effect could instead be explained by participants’ SES. Both SES (Hecht and Close, 2002; Hoff, 2003; Peterson and Pennington, 2015; Cheng and Wu, 2017) and the ART have been shown to correlate with measures of reading skill, so as a secondary analysis, SES was included as a possible predictor of individual differences. For each experiment, we ran a model including SES and verb bias as predictors without ART, followed by a final model including ART. Overall, we found no evidence that SES measures explained the observed correlation between the ART and pronoun comprehension (Experiments 1 and 2) or the observed correlation between ART and prediction (Experiment 3). A technical report (Johnson and Arnold, 2021) with the full analysis can be found with the Supplementary Materials at http://arnoldlab.web.unc.edu/publications/supporting-materials/johnson-arnold/.

Our study contributes to our understanding of how language experience relates to language processing. This study is the first to show that print exposure correlates with implicit cause biases at an individual level. This joins a growing set of findings about how referential processing is influenced by individual differences, which together show that print exposure changes the way language is processed at the discourse level.

## Data Availability Statement

The datasets presented in this study can be found in online repositories. The names of the repository/repositories and accession number(s) can be found at: https://osf.io/fuzp2/.

## Ethics Statement

The studies involving human participants were reviewed and approved by the Institutional Review Board at UNC- Chapel Hill. The participants provided their informed consent to participate in this study.

## Author Contributions

EJ: conceptualization, investigation, data collection, writing – original draft, and formal analysis. JA: conceptualization, methodology, writing – review and editing, supervision, and funding. All authors contributed to the article and approved the submitted version.

## Conflict of Interest

The authors declare that the research was conducted in the absence of any commercial or financial relationships that could be construed as a potential conflict of interest.

## Publisher’s Note

All claims expressed in this article are solely those of the authors and do not necessarily represent those of their affiliated organizations, or those of the publisher, the editors and the reviewers. Any product that may be evaluated in this article, or claim that may be made by its manufacturer, is not guaranteed or endorsed by the publisher.

## Footnotes

^1^The ART slope by items was removed from this model because it would not converge.

^2^The ART slope by items was removed from this model because it would not converge.

^3^However, the relative frequency of re-mentioning implicit causes may be restricted to only sentences with two human referents; for further discussion see Guan and Arnold (2021).
